# Four-month operational heat acclimatization positively affects the level of heat tolerance 6 months later

**DOI:** 10.1038/s41598-020-77358-7

**Published:** 2020-11-20

**Authors:** Alexandra Malgoyre, Julien Siracusa, Pierre-Emmanuel Tardo-Dino, Sebastian Garcia-Vicencio, Nathalie Koulmann, Yoram Epstein, Keyne Charlot

**Affiliations:** 1 Département Environnements Opérationnels, Unité de Physiologie de L’Exercice Et Des Activités en Conditions Extrêmes, Institut de Recherche Biomédicale Des Armées, 1 place Général Valérie André, 91223 Brétigny-sur-Orge, France; 2 LBEPS, Univ Evry, IRBA, Université Paris Saclay, 91025 Evry, France; 3 Ecole du Val-de-Grâce, 1, place Alphonse Laveran, 75230 Paris Cedex 5, France; 4 Sheba Medical Center, Heller Institute of Medical Research, 52621 Tel Hashomer, Israel

**Keywords:** Physiology, Medical research

## Abstract

Benefits obtained after heat acclimation/acclimatization should be completely lost after an estimated period of 6 weeks. However, this estimate is still hypothetical. We evaluate the long-term effects of heat acclimatization on the level of heat tolerance. Physiological and subjective markers of heat tolerance were assessed during a heat stress test (HST: 3 × 8-min runs outdoors [~ 40 °C and 20% RH] at 50% of their estimated speed at VO_2max_) performed on the 2nd day upon arrival to the desert military base in the United Arab Emirates after a first day of mostly passive exposure to heat. Among the 50 male French soldiers, 25 partook in a 4-month military mission in countries characterized by a hot environment ~ 6 months prior to the study (HA). The other 25 participants were never heat acclimatized (CT). Rectal temperature (*p* = 0.023), heart rate (*p* = 0.033), and perceived exertion (*p* = 0.043) were lower in the HA than CT group at the end of HST. Soldiers who experienced a former 4-month period of natural heat acclimatization very likely had a higher level of heat tolerance during exercise in the heat, even 6 months after returning from the previous desert mission, than that of their non-acclimatized counterparts.

## Introduction

A period of 10 days may be sufficient to show complete phenotypic heat acclimation (in an artificial environment) or acclimatization (in a natural environment), as determined by basic psycho-physiological parameters (decreases in core and skin temperature, heart rate (HR), sweat osmolality, thermal discomfort, and perceived exertion and increases in sweat rate)^1–3^. Such adaptations lead to improved heat tolerance and performance to exercise-induced heat stress.

Such physiological adaptations, however, appear to be transient^4,5^. In a recent meta-analysis, Daanen et al*.* estimated that improvements in heart rate (HR), core temperature (T_c_), and sweat rate decrease by ~ 2.5% d^−1^ once individuals are removed from the heat, suggesting that heat tolerance returns to baseline values between 5 and 7 weeks following the process of heat acclimation/acclimatization^5^. Nonetheless, these benefits are more rapidly recovered during the decay period, meaning that fewer days of heat re-acclimation are required to reach the same level of improvement^5–8^.

A more in-depth analysis of the decay response has shown that (1) large inter-study variability is likely explained by the diversity in protocols, (2) physical activity and/or environmental conditions during decay may be difficult to standardize, and (3) studies assessing decay after 25 days are few and inconclusive^5^. Moreover, heat re-acclimation has only been studied during incomplete decay, leading the authors to partially explain this rapid recovery by the fact that adaptations to heat were mostly retained when participants were re-exposed to heat^5^. Thus, the time required to reach complete decay is only theoretical and there are no known unequivocal characteristics. Indeed, re-induction after complete decay has never been assessed in humans. Furthermore, most studies relate to acclimation and less is known about acclimatization.

Elite athletes^5,9^ and certain professionals (soldiers, workers, engineers, rescue workers, and humanitarians, among others)^7,10–12^ may face several mid- to long-term competitions/missions in areas with a hot climate during their careers. Based on the aforementioned current knowledge, it appears that previous heat acclimation/acclimatization experiences are unlikely to influence the level of heat tolerance at the beginning of a repeated period of prolonged heat exposure if they are separated by more than 5–7 weeks. Nevertheless, such potential retention and/or “physiological memory” has never been directly assessed and we still do not know whether operational readiness in the heat is aided by past episodes of heat acclimation/acclimatization.

Studies on the effects of heat on soldiers may be very useful for addressing these questions. We, therefore, reanalyzed two sets of data on French soldiers^13,14^. Almost half of the participants were deployed for 4 months in countries characterized by a hot climate ~ 6 months before the study (usual period between two missions), which we considered as the heat acclimatized group (HA). Their psycho-physiological responses were compared during a heat stress test (HST) in a desert-like environment (~ 40 °C and ~ 20% of relative humidity) to those of a control group (CT), consisting of soldiers who had never been heat acclimatized during their professional life. Although this protocol was not originally designed to specifically study long-term adaptive remnants of heat acclimatization, it was used retrospectively to evaluate the long-term adaptive physiological changes acquired during a 4-month period of heat acclimatization. We hypothesized that the HA soldiers, although not exposed to heat for 6 months, would be more tolerant to heat than their counterparts from the same functional unit who were included in the CT group.

## Results

**Figure 1:**
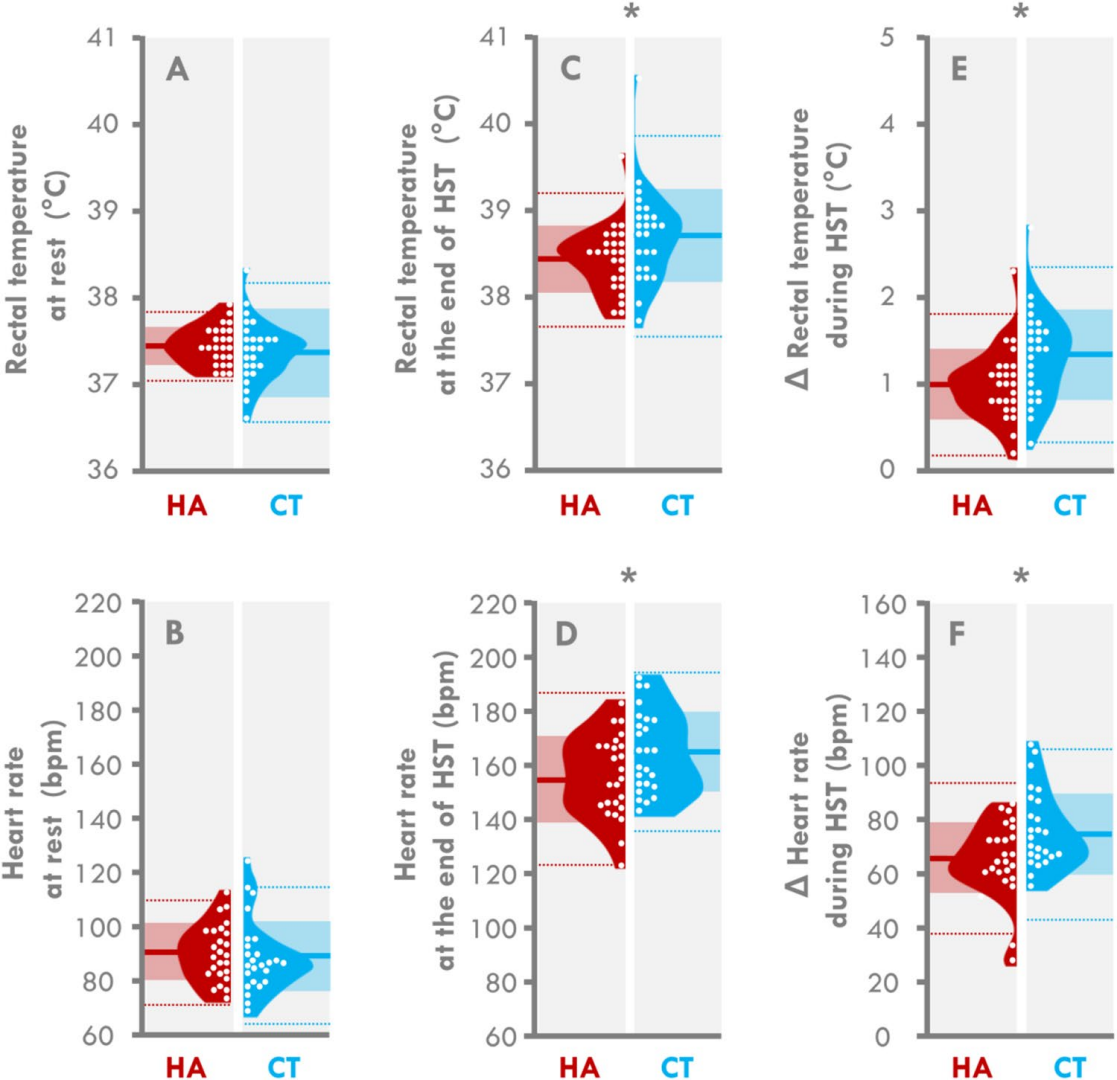
Violin plots of physiological variables during the heat stress test (HST). Individual data are represented by white dots and the mean by bold lines. Clear colored areas depict the SD. Dotted lines depict ± 2SD. HA: participants who were heat acclimatized 6 months before the experiment, CT: participants who were never previously heat acclimatized. *different from the HA group (*p* < 0.05).

At rest, there was no difference between the two groups for either T_rec_ (37.4 ± 0.2 vs 37.4 ± 0.4 °C for HA and CT, respectively, *p* = 0.421, *ES* = − 0.230) or HR (91 ± 10 vs 89 ± 13 bpm for HA and CT, respectively, *p* = 0.290, *ES* = − 0.176) (Fig. 1A,B, respectively).

At the end of the HST, the T_rec_ and HR were lower in the HA than CT group (T_rec_—38.4 ± 0.4 vs 38.7 ± 0.6 °C for HA and CT, respectively, *p* = 0.023, *ES* = 0.572, and HR—156 ± 15 vs 165 ± 15 bpm for HA and CT, respectively, *p* = 0.033, *ES* = 0.621, Fig. 1C,D, respectively). The mean differences between the two groups were 0.27 °C (95% CI − 0.01 to 0.54) for T_rec_ and 9.3 bpm (95% CI 0.8 to 17.8) for HR.

During the HST, the ΔT_rec_ was 25% lower in the HA than CT group (1.0 ± 0.4 vs 1.3 ± 0.5 °C for HA and CT, respectively, *p* = 0.015, *ES* = 0.710, Fig. 1E) and the ΔHR was 15% lower (65 ± 14 vs 76 ± 14 bpm for HA and CT, respectively, *p* = 0.016, *ES* = 0.805, Fig. 1F). The mean differences in the increase in T_rec_ and HR during the HST between the two groups were 0.34 °C (95% CI 0.07 to 0.61) and 11.4 bpm (95% CI 3.3 to 19.4), respectively.

**Figure 2:**
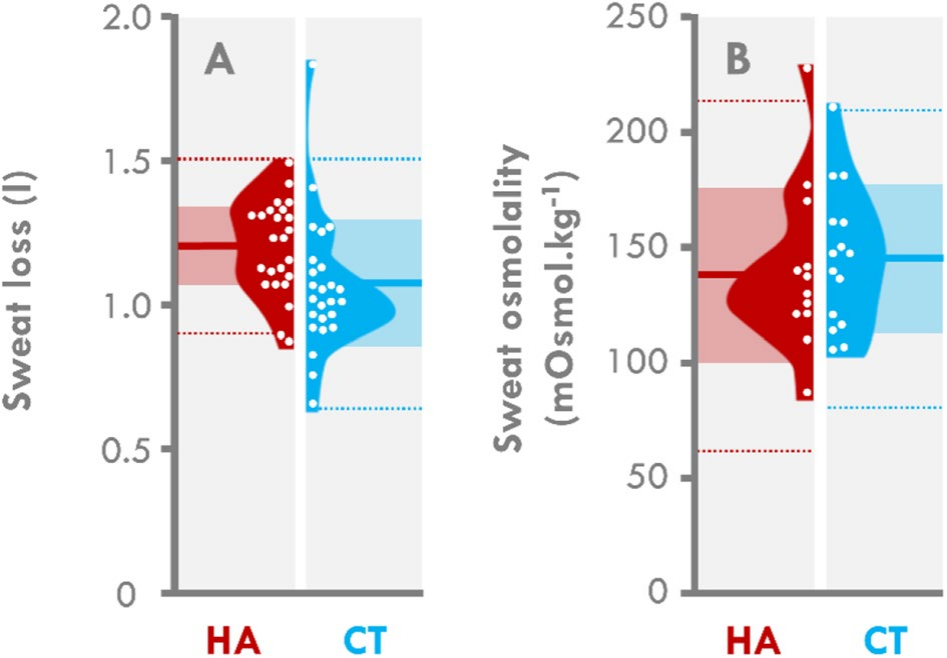
Violin plots of sweat rate and osmolality after the heat stress test (HST). Individual data are represented by white dots and the mean by bold lines. Clear colored areas depict the SD. Dotted lines depict ± 2SD. HA: participants who were heat acclimatized 6 months before the experiment, CT: participants who were never previously heat acclimatized.

Neither sweat loss (1.19 ± 0.16 L vs 1.05 ± 0.23 L for HA and CT, respectively, *p* = 0.231) nor sweat osmolality (140 ± 37 vs 144 ± 31 mOsmol kg^−1^ for HA and CT, respectively, *p* = 0.756, *ES* = 0.121) were different between the groups (Fig. 2A,B, respectively). Nevertheless, the larger difference in sweat loss in the HA group was considered to be moderate (*ES* = − 0.682).

**Figure 3:**
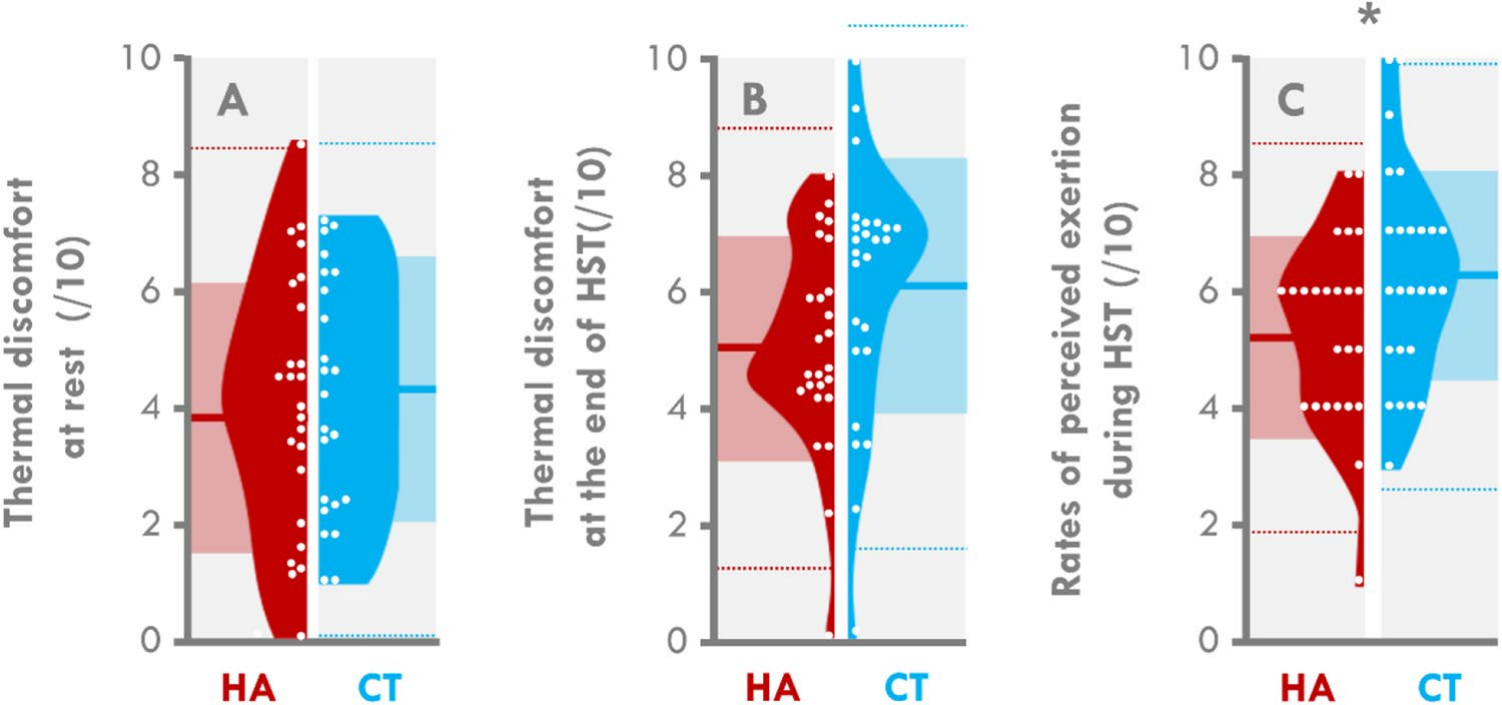
Violin plots of subjective variables during the heat stress test (HST). Individual data are represented by white dots and the mean by bold lines. Clear colored areas depict the SD. Dotted lines depict ± 2SD. HA: participants who were heat acclimatized 6 months before the experiment, CT: participants who were never previously heat acclimatized. *different from the HA group (*p* < 0.05).

Thermal discomfort at rest (3.8 ± 2.4 vs 4.1 ± 2.2 for HA and CT, respectively; *p* = 0.591, *ES* = 0.159) and at the end of the HST (5.1 ± 1.8 vs 6.1 ± 2.2 for HA and CT, respectively; *p* = 0.077, *ES* = 0.497), were no different between the two groups (Fig. 3A,B, respectively). The RPE was 15% lower in the HA than CT group (5.3 ± 1.6 vs 6.3 ± 1.9 for HA and CT, respectively, *p* = 0.043; Fig. 3C, *ES* = 0.545). The mean difference between the two groups was 0.95 points (95% CI − 0.04 to 1.94).

**Figure 4:**
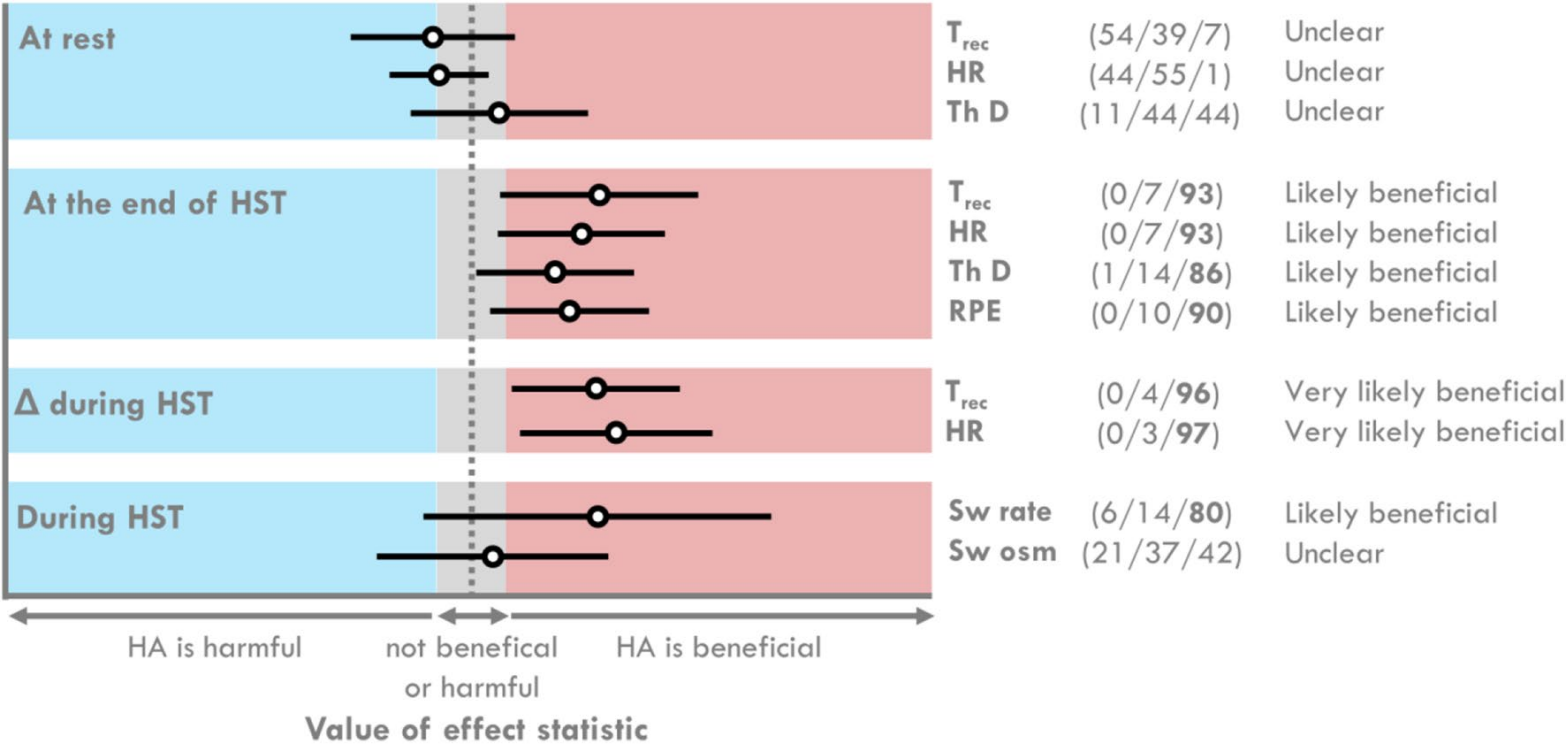
Confidence intervals (90%) and associated inferences for the differences between the heat acclimatization (HA) and control (CT) groups during the heat stress test (HST). The likelihood that previous HA is beneficial, trivial, or harmful was quantified using *p* values, value of effect statistics (mean differences between groups), degrees of freedom, and the smallest worthwhile change (0.2 × pooled SDs) with the following scale: 25–75%, possibly; 75–95%, likely; 95–99.5%, very likely; and ≥ 99.5%, most likely. T_rec_: rectal temperature, HR: heart rate, Th D: thermal discomfort, RPE: rates of perceived exertion, Sw: sweat, osm: osmolality.

All magnitude-based inferences are shown in Fig. 4. Overall, the HA group likely benefited from a reduction in T_rec_, HR, thermal discomfort, and RPE at the end of the HST and from an increase in sweat loss during the HST. Moreover, the HA group very likely had a smaller increase in T_rec_ and HR during the HST than the CT group.

## Discussion

Our results show that previously heat-acclimatized soldiers likely present better tolerance to exercise-induced heat stress, even after a de-acclimatization period of ~ 6 months, than their counterparts who had never been heat acclimatized. This suggests that a previous experience in the heat may increase the level of future heat tolerance and, therefore, enhance operational readiness at the beginning of a subsequent period of heat exposure. Two hypotheses could explain this observation: (1) the unusually long period of heat acclimatization (4 months instead of the 5 to 15 days commonly used in heat-acclimation studies and/or (2) the passive/active first day of heat exposure on the preceding day to the HST acted as a trigger, enabling rapid re-acclimation. We discuss both potential explanations below.

In the present study, the de-acclimatization period was long. Nonetheless, the physiological responses (T_rec_ and HR) to exercise performed outdoors at ~ 43 °C and ~ 25% relative humidity and RPE were very likely favorable in the HA group relative to the CT group. This suggests that decay may not have been complete, despite the long elapsed time without exposure to environmental heat. The analysis of short-term acclimation revealed that most^6,15^, but not all^7^, studies showed that improved HR returned to baseline values between 17 and 21 days after acclimation. Similar decay kinetics was also found for T_rec_^16,17^. The latter, however, was less systematic, as most studies reported only a partial decay at the end of the observation period^6,7,15^. Improvements in sweat rate systematically diminished after ~ 20 days^5^, whereas those for subjective markers (RPE or thermal sensations) were retained after 16 to 26 days^8,18,19^. Nevertheless, the results from several studies with similar or longer periods of decay highlight large variability in these decay responses^8,16,18,19^. According to Weller et al., re-exposure to heat after 26 days of heat-acclimation decay showed that the improvements in T_rec_ and HR were almost completely preserved (0 and 26% decay, respectively)^8^. For reasons that are difficult to clearly identify (differences in the design of the HST, environmental conditions of acclimation, participants, etc.), decay responses may vary highly between studies. Therefore, it is difficult to estimate the duration required to reach complete decay.

Of note, in contrast to our observations, a recent study of Corbett et al*.* showed that a 10-day heat acclimation protocol induced no observable effect on heat adaptations during a subsequent period of heat acclimation 3 to 18 months later^20^. Apart from this study, no other attempt has been made to assess the heat tolerance status after long periods of acclimatization decay. However, many aspects of our heat-acclimatization study protocol differed from those of other heat-acclimation studies that assessed the decay of heat adaptation. First, our participants were acclimatized for 4 months, whereas acclimation under laboratory-controlled conditions lasted ~ 10 days (from 4 to 14 days) in most decay studies^6–8,15–20^. Second, although difficult to estimate, our participants spent most of the day, and sometimes nights, outdoors in ambient temperatures of 30 to 50 °C. In classical heat acclimation protocols, heat exposure only occurs during the exercise session (up to 120 min d^−1^)^6–8,15,16,18,20^. Finally, light sports clothing (shorts, with or without a tee-shirt) are usually worn during heat-acclimation sessions. Our participants had to wear military outfits (mostly battledress) while exposed to the heat, which enhances the thermal burden. Thus, it is likely that the thermal stress lasted longer (given both the duration of acclimatization and daily exposure) and was harsher (given the reduction in heat loss induced by the clothing) than in most other studies. It follows that the conditions in the present study may have induced more sustainable adaptations than those of a classical short-duration heat-acclimation program. This may be corroborated by studies in an animal model that showed differences between short- and long-duration acclimation period^21,22^ and studies in humans showing that hematological adaptations (increase in hemoglobin mass) may be obtained only after long-term heat acclimations (5 weeks)^23,24^. Nevertheless, it has never been directly assessed whether the single or combined differences of such acclimatization could affect the retention of heat-acclimatization benefits. The meta-analysis of Daanen et al.^5^ suggested that the duration of heat acclimatization may be a factor involved in the kinetics of decay, at least for the decay of T_rec_.

An alternative or concomitant hypothesis is rapid heat re-acclimation. At least 24 h elapsed between landing in the UAE and the HST, during which participants were mostly passively exposed to heat (information meetings in rooms without air conditioning) but also performed certain short physical activities (handling and carrying luggage). We hypothesize that this exposure may have been sufficient to trigger rapid re-acclimation, observable during the HST performed the day after. This is in accordance with a previous study, which showed that T_rec_ and HR during exercise reach post-acclimation values more rapidly after incomplete decay than during the first acclimation period^5^. Furthermore, Stephen and Hoag estimated that heat re-acclimation is eight times stronger for HR and 12 times stronger for T_rec_ during the decay period than during the heat acclimation period^6^. Thus, two days of re-acclimation are theoretically sufficient to restore the benefits obtained after 16 and 24 days of heat acclimation, respectively. Such faster apparent re-induction of acclimation must be tempered by the fact that heat re-exposure always occurred before complete decay in all studies that proposed this hypothesis. Adaptations were therefore partially retained and maximal improvements were automatically more rapidly obtained during the re-induction period^5^. Thus, re-acclimation has never been assessed after complete decay in humans and we still do not know whether re-acclimation is in fact faster than a former acclimation process. Animal studies led by the laboratory of Horowitz have provided mechanistic evidence to support faster re-acclimation^21^. They demonstrated that genes important for cellular protection, such as *Bcl-xL*, an anti-apoptotic gene, and the *Hspa1a* and *Hsp90aa1* genes (encoding the proteins Hsp70 and Hsp90, respectively), remained upregulated in the cardiac tissue of rats throughout de-acclimation, provided that the initial period of heat exposure and acclimatization had been sufficient^22^. Such acclimatory memory appears to be promoted by epigenetic changes, particularly through modifications of the chromatin state^25^. Such a “dormant” state predisposes the chromatin to re-induction of gene expression when re-exposed to heat and ,subsequently, rapid activation of the cytoprotection profile, possibly responsible for more rapid physiological re-acclimation, in as little as one or two days. Although we were unable to measure aspects of either cellular tolerance or epigenetic modifications in the present study, it cannot be ruled out that such molecular alterations contributed to our observations. The long duration of initial heat acclimation in our study (several months), the time course of de-acclimation, and the timing of our HST, performed one day after arrival to a hot region, are consistent with epigenetic changes, which require long-term acclimation and a short time for re-induction^22,26^. Although it is not possible to determine whether remaining heat tolerance due to only partial decay or quick re-induction are responsible for these observations, these two mechanisms are not contradictory and could synergize or concern different variables.

Our study had several limitations. First, we were unable to control the conditions of the heat acclimatization period. Given the location and type of mission, the heat acclimatization conditions were not strictly identical between participants. However, operational missions that are characterized by overall moderate to high levels of daily physical activity performed outdoors under hot conditions are sufficiently physiologically stressful to induce heat acclimatization^9,27^. In this military context, we have already shown that heat acclimatization is almost completely achieved after 15 days and that the improvement between day 10 and day 15 is lower than that between day 0 and day 10^13,14^. Thus, we considered these soldiers, who were engaged in a 4-month deployment in hot countries, to be fully acclimatized. Second, the design of this study precluded a clear assessment of decay (modification of psychophysiological markers between the end of the 4-month mission and the beginning of the following mission 6 months later). Nevertheless, participants from both groups originated from the same four regiments and therefore performed the same professional physical activities in the same environments during the 6-month period preceding the HST. Moreover, the proportion of soldiers for the four included regiments was well balanced between the two groups (regiment #1: 7/7–#2: 10/6–#3: 5/6–#4: 3/6 in the HA and the CT groups, respectively). It is therefore highly unlikely that differences between groups depicted in this article were due to differences in environmental and/or exercise-induced thermal strain. Nevertheless, it cannot be excluded that such regular professional physical activity during the 6-month period participated in the partial retention of heat acclimatization, although it was not sufficient to induce de novo heat adaptation, given that it coincided with the winter-spring seasons in France. Finally, the level of heat tolerance was different between soldiers with the same profile but with one group having experienced a recent mission in the heat. The reporting of this result is necessary to motivate further experimentation to better characterize the long-term decay of heat acclimation, which is currently a neglected topic.

Soldiers who performed long-term (4 months) operational missions in a hot environment, and thus considered to be heat acclimated, were likely more tolerant to a HST performed 6 months after returning to a temperate climate than soldiers who had never been heat acclimatized. Such improved acute heat tolerance was characterized by lower T_rec_ and HR at the end of a HST, smaller absolute increases in T_rec_ and HR during a HST, and lower RPE for the HA than CT group. It is still not possible to clearly determine the mechanisms involved (long-term remnant of adaptation and/or rapid re-induction). However, if confirmed, these results show that individuals with a history of long-term heat acclimation/acclimatization may be more tolerant during the first days of re-exposure to heat (even 6 months after the acclimatization period) than their non-acclimated counterparts. In a professional context, this may help in selecting individuals with greater operational readiness to perform delicate tasks in the heat. Although this recommendation requires direct experimental support, we recently observed that individuals starting a heat acclimatization period with a deficit in heat tolerance did not manage to catch up those who were originally more heat tolerant after 15 days^28^.

## Methods

### Participants

The study consisted of French Army soldiers who participated in our previous studies^13,14^. These studies were performed at the request of the French Armed Forces in the United Arab Emirates (UAE) and approved by the scientific leadership of the French Armed Forces Biomedical Research Institute. This study required no invasive measurements and did not impose unfamiliar tasks to the participants. In this case, we were exempted according to the Institute regulation to obtain an ethical approval from a civilian Committee as long as the experiment was realized in accordance with the Declaration of Helsinki. All participants were found to be healthy by military physicians and were briefed before leaving France, during which they were then informed of the benefits and risks of the investigation prior to giving their written informed consent, in accordance with the Declaration of Helsinki.

The study was conducted in two steps (in May–June 2016 and 2017) and the soldiers did not participate in any mission (in France or elsewhere) where the climate could have been considered to be hot (dry or humid) for the previous 6 months. The location, period, and date of the last mission in a hot country were noted (if applicable). Participants who took part in a former 4-month military mission in countries characterized by a hot environment ~ 6 months (between 5 and 7 months) before the study were included in the HA group. This 6-month period is generally the minimal mandatory period separating two missions. Participants who were deployed less than 5 months or more than 7 months after their previous missions or who were unable to provide accurate information were excluded from this retrospective analysis. Participants who travelled for personal reasons to countries characterized by a hot environment during the previous 8 months were also excluded from the study. Finally, from a total of 120 soldiers who were screened for the study, 90 were first included—32 in the HA group and 58 in the CT group. However, using Student’s t test, we found that the groups were not balanced in terms of age (*p* < 0.001) or fitness level (*p* = 0.005), based on their last Cooper 12-min run test performance^29^ (a test routinely used by the French Army to annually assess the level of aerobic fitness) performed during the month before departure to the UAE in a temperate environment. The differences observed between the HA and CT group are inherent to French military career management. New recruits are allowed to participate in foreign operational missions only after at least 1 year of military training and assimilation. Participants in the HA group were deployed for at least the second time, implying that they had been in military service for at least 3 years. Consequently, they were older and more highly trained than the participants in the CT group, which may explain the difference in the performance of the Cooper test. We therefore removed the oldest participants from the HA group (> 32 years) and the youngest (< 21 years) and less fit participants from the CT group (< 2,500 m). Twenty-five participants were finally selected for each group. A Figure available in supplementary files presents the repartition of the conserved and removed participants for each outcome presented in this article. The participant characteristics in each group are summarized in Table 1. The mean duration between the study and the last mission in the HA group was 6.0 ± 0.6 months.

**Table 1:** Participant characteristics.

	Heat acclimatized (HA)	Control (CT)
n	25	25
Age (y)	25.1 ± 2.6	24.1 ± 1.9
Height (cm)	177 ± 6	177 ± 5
Weight (kg)	74.4 ± 6.9	75.5 ± 11.7
Body mass index (kg m^−2^)	23.9 ± 2.0	23.9 ± 3.0
Body surface area (m^2^)	1.91 ± 0.11	1.92 ± 0.16
Cooper performance (m)	2920 ± 173	2890 ± 151
Estimated VO_2max_	53.9 ± 3.8	53.0 ± 3.5
Running speed (km h^−1^)	7.7 ± 0.5	7.5 ± 0.4

Means ± SD.

VO_2max_: maximal oxygen uptake.

### Procedure

Approximately 6 months before the beginning of the study, participants in the HA group carried out 4 months operational heat acclimatization. It took place in countries classified (according to the Köppen–Geiger climate classification system) as (1) tropical savanna climates with a dry-winter: Central African Republic (n = 10; mean day and night temperature = 27.3 °C, mean day and night relative humidity = 63%, mean day and night indoor WBGT temperature = 23.8 °C) and Ivory Coast (n = 3; 28.1 °C, 89%, 24.5 °C), (2) hot desert climates: Mali (n = 3; 30.5 °C, 20.3%, 20.9 °C) and Djibouti (n = 2; 28.8 °C, 71.3%, 26.3 °C), 3) tropical rainforest climates: New-Caledonia (n = 5; 26.4 °C, 78%, 21.0 °C), and 4) hot-summer Mediterranean climates: Lebanon (n = 2; 23.1 °C, 71.3%, 20.7 °C). For comparison, the CT group remained in France in a temperate oceanic climate (n = 25; 12.5 °C, 71.7%, 10.3 °C).

**Figure 5:**
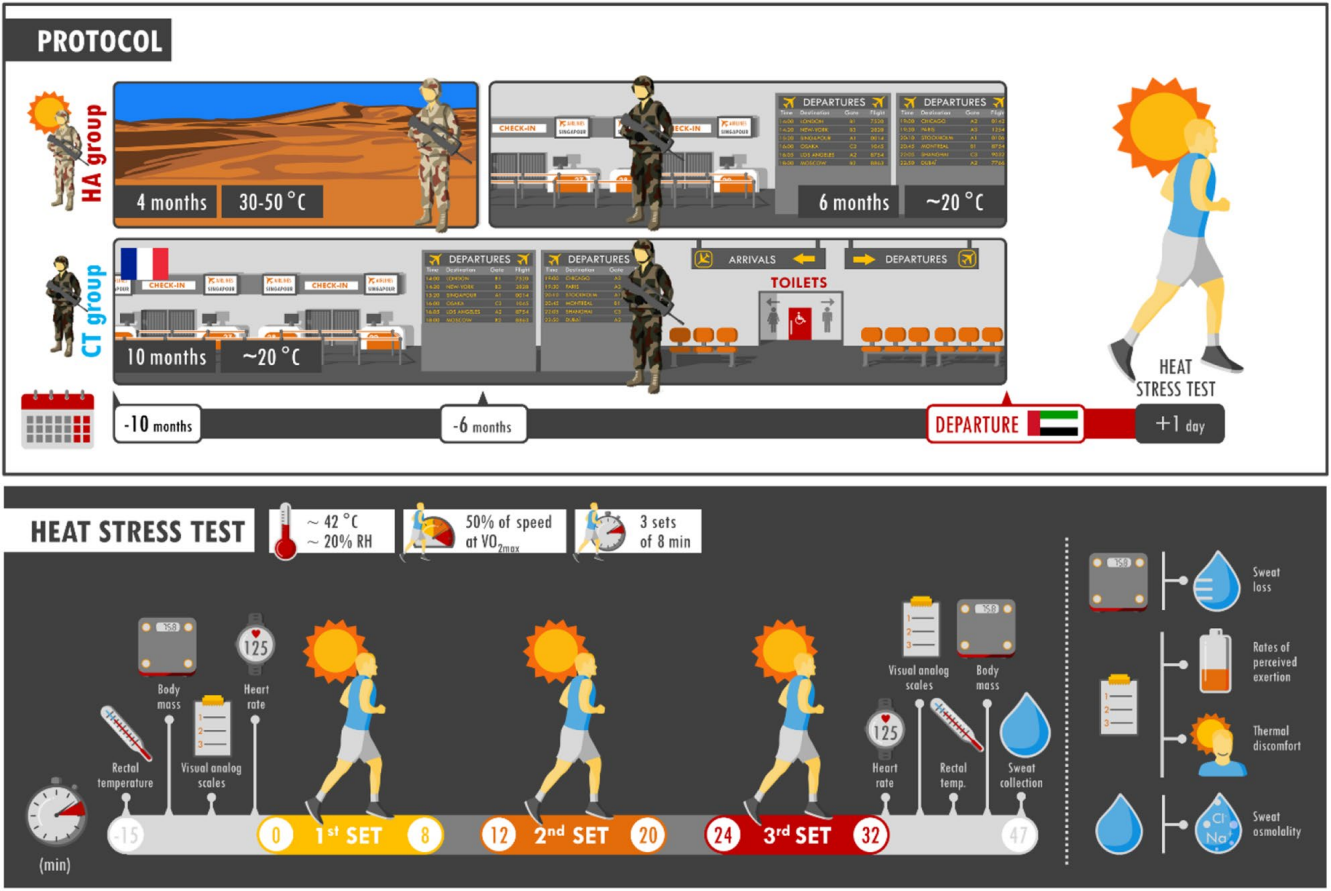
Description of the protocol. Participants in the previously heat-acclimatized group (HA) performed a 4-month operational mission in countries with hot climate 6 months before the experiment, whereas the control group (CT) did not. All participants were stationed in France for 6 months before the experiment. The airport background symbolizes their main mission in France consisting in protecting public places. One day after their arrival in the UAE, participants performed a heat stress test (HST) (see text for further details).

The HST has been extensively described in our previous publications^13,14^ and is presented in Fig. 5. The day after the soldiers arrived in the UAE they were mostly passively exposed to the environmental heat (mostly a camp visit and information meetings) and performed short physical activities (luggage handling and carrying). On the following day, they performed a HST (three 8-min runs, outdoors, at 50% of their estimated speed at VO_2max_). Running intensities were calculated from the running speed at maximal oxygen uptake (VO_2max_) calculated from the Cooper 12-min run test results^29^.

As explained in our previous article^26^, environmental conditions were measured from the beginning to the end of each session with a weather meter (Kestrel Meter 440 Heat Stress Meter, Birmingham, MI, USA) near the track at a height of 1.2 m and exposed directly to the sun. During the sessions, the mean dry bulb temperature was 42.6 ± 3.2 °C, relative humidity 25.4 ± 11.6% (wet-bulb temperature 27.3 ± 2.2 °C), globe temperature 56.0 ± 3.2 °C, WBGT 34.6 ± 1.6 °C, and wind speed 1.7 ± 0.4 km h^−1^.

Rectal temperature (T_rec_), nude dry body mass, and HR were measured, and thermal discomfort assessed before and after exercise. Sweat osmolality was measured and the rate of perceived exertion (RPE) was assessed at the end of exercise. The test was divided into four daily sessions with 15 participants per session (two in the morning and two in the afternoon). The proportion of participants from the HA and CT groups was similar for each session.

### Measurements

Rectal temperature was measured by the participants themselves with electric thermometers (PX-TH 418, Pelimex, Ingwiller, France) at a depth of 6 cm. Recent studies have suggested that measurements at this depth are concordant with deeper measurements^30,31^. Participants were equipped with a chest belt and a HR monitor wrist receptor (RC3 GPS, Polar, Kempele, Finland). Resting HR was measured for 5 min just before the HST in an upright position, without moving, and the lowest 1-min plateau value was used for the mean calculation. The HR at the end of exercise corresponded to the mean of the last 30 s of the final 8-min run. Sweat loss was calculated from the nude dry body weight difference measured before and after the HST with a balance (Mettler Toledo ICS 425d, Greifensee, Switzerland, accurate to 20 g). Sweat was collected using a self-made impermeable sweat collector (10-cm square) placed on the chest and stored in 2-ml aliquots. Osmolality was assessed using a freezing point osmometer (Osmomat 3000 basic, Gonotec, Berlin, Germany).

For thermal discomfort, participants answered the question “How do you find the thermal environment?” by placing a horizontal dash on a vertical 10-cm scale, on which the bottom end represented “comfortable” and the top end “very uncomfortable”. The distance in centimeters between the lower extremity and the marked line depicted the thermal discomfort score. This scale was adapted from a previous study^32^ and translated into French. Rates of perceived exertion were assessed using a 0 to 10 scale^33^.

### Statistical analyses

After assessing whether the data were normally distributed or not (Shapiro–Wilk test), we either used Student’s t test (parametric test) or the Mann–Whitney U test (non-parametric test) to compare the participant characteristics and psychophysiological variables measured at rest and during the HST between the HA and CT groups. These differences were also examined using standardized differences, based on Cohen’s effect size (ES) principle: > 0.2 (small), > 0.5 (moderate), and > 0.8 (large)^34^. Although still debated^35,36^, the method of magnitude-based inferences^37^ provides a more nuanced and complementary approach of interpreting *p* values with respect to the smallest worthwhile effect. The likelihood that previous heat acclimatization is beneficial, not beneficial or harmful, or harmful was quantified using *p* values, value of effect statistics (mean differences between groups), degrees of freedom, and the smallest worthwhile change (0.2 × pooled SDs)^38,39^, with the following scale: 25–75%, possibly; 75–95%, likely; 95– 99.5%, very likely; and ≥ 99.5%, most likely^40^. Data in the text are presented as the means ± standard deviation (SD). Significance was defined as *p* < 0.05. Analyses were performed using SPSS software (v20, IBM SPSS Statistics, Chicago, IL, USA).

## Supplementary information

Supplementary Figure Legend.

Supplementary Figure.

## Data Availability

The datasets generated during and/or analysed during the current study are available from the corresponding author on reasonable request.
