# Supplementary material for: Four-month operational heat acclimatization positively affects the level of heat tolerance 6 months later
Source: Sci Rep. 2020 Nov 20;10:20260. doi: 10.1038/s41598-020-77358-7 (PMC7680124; doi:10.1038/s41598-020-77358-7)
Supplement: Supplementary file 1 — Supplementary Figure Legend. [file 41598_2020_77358_MOESM1_ESM.docx]

**Four-month operational heat acclimatization positively affects the level of heat tolerance six months later**

Alexandra Malgoyre, Julien Siracusa, Pierre-Emmanuel Tardo-Dino, Sebastian Garcia-Vicencio, Nathalie Koulmann, Yoram Epstein, and Keyne Charlot

**Legend of supplementary Figure - Psychophysiological markers measured during the heat stress test (HST).** Individual data are represented by dots and the mean by bold lines. Clear colored areas depict the SD. Filled dots represent Participants conserved in the final analysis

And empty ones those removed from the final analysis.
