# Supplementary figures and images for: Four-month operational heat acclimatization positively affects the level of heat tolerance 6 months later
Source: Sci Rep. 2020 Nov 20;10:20260. doi: 10.1038/s41598-020-77358-7 (PMC7680124; doi:10.1038/s41598-020-77358-7)

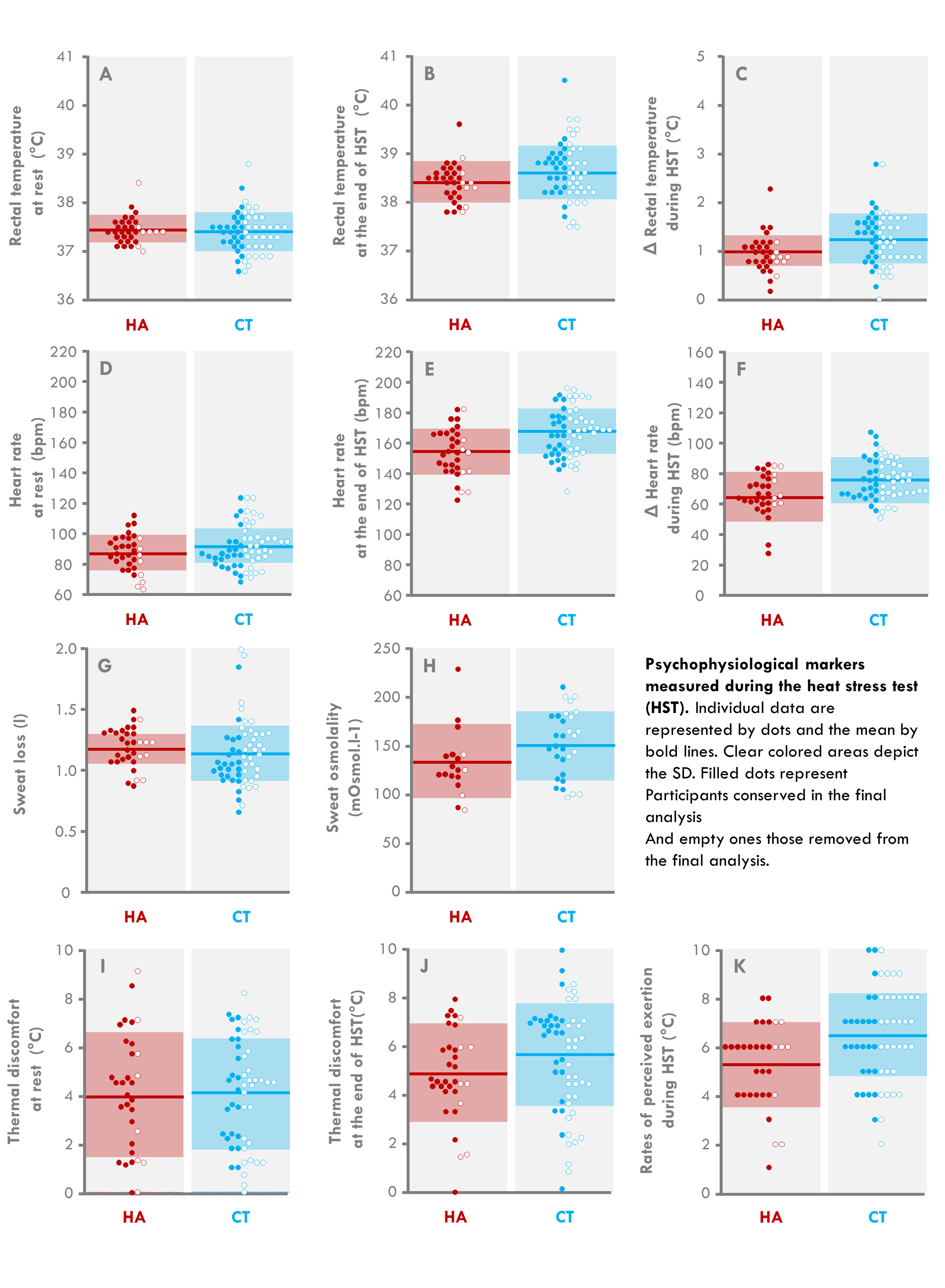

Supplement: Supplementary file 2 — Supplementary Figure. [file 41598_2020_77358_MOESM2_ESM.tif]
